# Ibrutinib response in a patient with refractory mixed essential cryoglobulinemia

**DOI:** 10.1002/jha2.686

**Published:** 2023-04-01

**Authors:** Haya Jamali, David Wu, Lorinda Soma, Michael Linenberger, Mark H. Wener, Lev Silberstein

**Affiliations:** ^1^ Department of Medicine Massachusetts General Hospital Boston Massachusetts USA; ^2^ Department of Laboratory Medicine and Pathology Seattle Washington USA; ^3^ Department of Pathology City of Hope Medical Center Duarte California USA; ^4^ Division of Hematology University of Washington Seattle Washington USA; ^5^ Division of Rheumatology University of Washington Seattle Washington USA; ^6^ Fred Hutchinson Cancer Center Seattle Washington USA

**Keywords:** cryoglobulinemia, ibrutinib, Waldenstrom macroglobulinemia

1

A 74‐year‐old male presented with fatigue, parasthesiae, Raynaud's phenomena, visual disturbances, and recurrent lower extremity rash (left). Investigations revealed an elevated serum cryoglobulin, rheumatoid factor >1320 IU/ml (ref. <15 IU/mL), undetectable C4, and C3 of 25 mg/dL (ref. 87–200 mg/dL). A small amount of IgM kappa monoclonal protein (M spike of 2.5%) and polyclonal IgG were identified by immunofixation. A diagnosis of mixed (Type II) cryoglobulinemia was made. Testing for Hepatitis B and C was negative. Positron emission/computed tomography (PET‐CT) showed no evidence of lymphoma. Although the bone marrow appearance raised suspicion for Waldenstrom macroglobulinemia (WM), testing for MYD88 L265P mutation, which is commonly associated with this disorder, was negative. Of note, scattered CD20‐positive B‐cells comprised only ∼5% of bone marrow cellularity.

The patient's rash gradually evolved into large ulcers over bilateral medial malleoli. Despite treatment with immunosuppression, rituximab, and plasmapheresis, they expanded to 9.3 × 8.9 × 0.3 cm on the left and 8.6 × 5.6 × 0.4 cm on the right (middle), requiring extensive use of opioids and significantly interfering with patient's mobility. A second bone marrow biopsy was again suggestive of WM. However, the frequency of abnormal B‐cells was even lower (1.7%), likely reflecting prior therapy. Therefore, B‐cells were purified by flow sorting using antibodies against CD19, CD20, and CD38 prior to molecular testing. Critically, this approach revealed the presence of MYD88 L265P mutation, **supporting** the diagnosis of WM and justifying therapy with Ibrutinib, a Bruton kinase inhibitor which is highly effective in this disease.

Starting Ibrutinib 420 mg daily was a clear turning point in the patient's clinical course. After just a few days, he reported a drastic improvement in fatigue and leg ulcer pain. Within the following 2 months, he became pain‐free and no longer required narcotics. By the end of the 9 months of therapy, the ulcers were completely healed (right) and quantitative IgM went down from 1058 to 325 mg/dL after the first 3 months. Nearly 2 years since starting the treatment, the patient is asymptomatic except for mild parasthesiae. He continues on Ibrutinib at a lower dose (280 mg daily).

Cryoglobulinemia (CG) is a rare multisystem disorder which is caused by immunoglobulins with a capacity to aggregate at lower temperatures (cryoglobulins). Notably, even a small amount of cryoglobulin in the serum is sufficient to cause disease. Hence, a low‐burden lymphoproliferative disorder that serves as the source of monoclonal cryoglobulin may be difficult to diagnose, precluding the use of targeted therapies. In agreement with a single prior report [1], our case shows that Ibrutinib can be remarkably effective in refractory CG due to WM. Unexpectedly, we found that despite a near‐complete resolution of symptoms with Ibrutinib, the patient's cryoglobulins/IgM paraprotein persisted. In addition, the rheumatoid factor was still elevated, and C4 complement remained undetectable. This suggests that the therapeutic benefit of Ibrutinib may be explained not only by anti‐tumor effect but also anti‐inflammatory properties, as demonstrated by its efficacy in COVID [2], graft‐*versus*‐host disease [3], and autoimmune conditions [4]. Finally, our case shows that enriching for B‐cells prior to molecular analysis may aid the diagnosis of occult lymphoproliferative disorder in other patients, for example those with IgM monoclonal gammopathy of clinical significance, who may also benefit from a trial of targeted therapy.

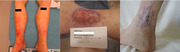

## AUTHOR CONTRIBUTIONS

H. Jamali wrote the manuscript. D. Wu performed cell sorting and molecular analysis. L. Soma performed morphological assessment of the bone marrow. M. Linenberger contributed to clinical management of the patient (plasma exchange). M. Wener provided rheumatology expertise and co‐managed the patient with LS. L. Silberstein provided haematology expertise, co‐managed the patient with MW, and wrote the manuscript.

## FUNDING INFORMATION

The authors received no specific funding for this work.

## CONFLICT OF INTEREST STATEMENT

None of the authors received financial compensation from an external source in return for writing or publishing this paper.

## ETHICS STATEMENT

The patient consented to the use of their clinical data and photographs for this correspondence.
